# Facile Synthesis of Multifunctional Magnetoplasmonic Au-MnO Hybrid Nanocomposites for Cancer Theranostics

**DOI:** 10.3390/nano12081370

**Published:** 2022-04-16

**Authors:** Cong Tian, Zhe Tang, Yike Hou, Asim Mushtaq, Shafaq Naz, Zhangsen Yu, Jabeen Farheen, Muhammad Zubair Iqbal, Xiangdong Kong

**Affiliations:** 1 Institute of Smart Biomedical Materials, School of Materials Science and Engineering, Zhejiang Sci-Tech University, Hangzhou 310018, China; 201920301046@mails.zstu.edu.cn (C.T.); 202030302183@mails.zstu.edu.cn (Z.T.); 202110301007@mails.zstu.edu.cn (Y.H.); asim_college@yahoo.com (A.M.); jabeenfarheen87@zstu.edu.cn (J.F.); 2 Zhejiang-Mauritius Joint Research Center for Biomaterials and Tissue Engineering, Hangzhou 310018, China; 3 Department of Mathematics, University of Gujrat, Hafiz Hayat Campus, Gujrat 50700, Pakistan; drshafaq.naz@uog.edu.pk; 4 Laboratory of Nanomedicine, Medical Science Research Center, School of Medicine, Shaoxing University, Shaoxing 312000, China; yzs@usx.edu.cn

**Keywords:** plasmonic nanoparticles, magnetic nanocomposites, T Magnetic contrast agents, photothermal therapy, cancer theranostics

## Abstract

Significant attention is paid to the design of magnetoplasmonic nanohybrids, which exploit synergistic properties for biomedical applications. Here, a facile method was employed to prepare plasmonic magnetic Au-MnO heterostructured hybrid nanoparticles for imaging-guided photothermal therapy of cancers in vitro, with the view to reducing the serious drawbacks of chemotherapy and gadolinium-based contrast agents. The biocompatibility of the prepared Au-MnO nanocomposites was further enhanced by Food and Drug Administration (FDA)-approved triblock copolymers Pluronic^®^ F-127 and chitosan oligosaccharide (COS), with complementary support to enhance the absorption in the near-infrared (NIR) region. In addition, synthesized COS-PF127@Au-MnO nanocomposites exhibited promising contrast enhancement in T_1_ MR imaging with a good r_1_ relaxivity value (1.2 mM^−1^ s^−1^), demonstrating a capable substitute to Gd-based toxic contrast agents. In addition, prepared COS-PF127@Au-MnO hybrid nanoparticles (HNPs) produced sufficient heat (62 °C at 200 μg/mL) to ablate cancerous cells upon 808 nm laser irradiation, inducing cell toxicity, and apoptosis. The promising diagnostic and photothermal therapeutic performance demonstrated the appropriateness of the COS-PF127@Au-MnO HNPs as a potential theranostic agent.

## 1. Introduction

Promising scientific efforts have been made in the realm of nanotechnology during the last two decades, driving effective treatments for life-threatening diseases. Nanoparticle-based therapeutics agents with controlled dimensions (1–100 nm) make an outstanding contribution to biomedical applications owing to their unique characteristics such as surface functionalities, choice of sizes, and manipulation abilities under various external stimuli [1,2]. The properties mentioned above, especially size, decide the administration pathway, for example, intravascular, oral or intratumoral [3,4]. Drugs, genes, therapeutic agents, and imaging moieties can be loaded onto the surface or insides of engineered NPs for personalized nanomedicine [5,6]. In addition, novel nanocomposites with controlled size have shown considerable advantages over individual materials due to multifunctional and enhanced properties because of their hybrid nature. Taking advantage of their multiple characteristics, these nanocomposites have been effectively employed to improve the life span of cancer patients [7,8,9].

Presently, the available cancer treatments are limited to radiation and chemotherapy, which wreak havoc on patients, resulting in severe pain, vomiting, nausea, hair loss, and severe infections [10]. Nanomaterials with targeting ligands demonstrate significant potential to overcome the side effects of conventional chemo-therapeutics drugs. Nanomaterials with small sizes offer high surface area, which enables loading and delivery of targeting agents efficiently [11]. With the advancement in therapeutic techniques, photothermal ablation (PTA) therapy has gained greater attention recently. Near-infrared (NIR) laser responsive photosensitizers generate sufficient heat to ablate tumors after successfully localizing into cancerous cells [12,13]. PTA is minimally invasive and, in some cases, a more effective treatment than surgery. Several kinds of photo-responsive agents, including indocyanine green [14,15], polyaniline [16], carbon-based materials [17], metal chalcogenide [18], and noble metal nanoparticles [19], have been receiving attention in phototherapy applications. 

However, organic-based photosensitizers often encounter some shortcomings such as easy light bleaching, low photothermal conversion efficiency, poor stability, complicated synthesis, and rapid clearance from the body [20]. In order to resolve these issues, inorganic nanogold has received promising interest to achieve high photothermal conversion efficiency with excellent biosafety owing to its unique plasmonic, acoustic, and electric properties, as well as multifunctionality endowed by its various dimensions and morphologies [21]. Photothermal ablation using Au-based NPs has been assessed in clinical trials for different cancers [22,23]. However, a high concentration of Au-NPs and the high power density of the lasers may increase the temperature within normal cells, resulting in damage to the entire tissue. Therefore, a combination of different therapies, such as photo-chemotherapy and photo-radiotherapy, possesses better outcomes due to synergistic behavior in a safer fashion [24,25]. Furthermore, in order to attain more effective PTT outcomes with minimum side effects, imaging modality coupled with a therapeutic agent is necessary for the active delivery of photothermal agents at the desired target [26,27,28]. Among medical diagnostics techniques, magnetic resonance imaging (MRI) is a commonly used noninvasive imaging tool in clinics, showing comprehensive real-time diagnosis with high spatial and temporal resolutions. Nevertheless, the low sensitivity (i.e., differentiation between normal tissues and lesions) of MRI is a major obstacle to the precise diagnosis, and additional contrast enhancement probes are required to improve the visibility [29,30]. In clinics, gadolinium-based contrast enhancement agents have been employed globally. However, serious safety fears arising from free Gd^3+^ ions in circulation, especially in the kidneys and brain, is worrying, and its retention develops nephrogenic systemic fibrosis (NSF), a fatal disease without any definite treatment. Therefore, scientists pay serious attention to investigating non-Gd replacements [31,32].

In addition, the combination of magnetic iron oxide and plasmonic gold has attained noticeable attention in nanomedicine, particularly multi-modal imaging and image-directed therapies. Magnetoplasmonic (Fe_3_O_4_@Au) nanocomposites demonstrated T_2_-weighted MR imaging, appearing dark, and showed good photothermal properties [33,34]. Fe_3_O_4_-decorated Au nanoparticles showed enhanced synergistic properties against cancerous cells [35]. However, iron oxide-based nano-contrast agents, for example clinically approved Feraheme (ferumoxytol), encountered some challenges, such as artifacts in MR signals commonly developed by metals that are hard to detect and distinguish, aggregation in vivo due to magnetic interactions under a magnetic field, fluctuating behavior in T_1_ and T_2_ signals, potentially life-threatening allergy, and chronic diseases. Research showed that these shortcomings could be overcome by various surface coatings [36,37,38,39,40].

Recently, manganese-based MRI nano-probes have emerged as a new class of efficient, positive contrast and potential alternatives of Gd^3+^ [41,42,43]. Paramagnetic Mn^2+^ ions induced a significant characteristic for shortening T_1_ relaxation times [44]. Au@MnO_2_ core/shell (50 nm) nanostructures were prepared by laser irradiation of Au NPs in the presence of KMnO_4_, which may hinder the photothermal properties after the second irradiation [45]. Moreover, some large-sized and unstructured Au-MnO_2_ particles have been reported to possess good properties for the detection of electrocatalytic hydrogen evolution, as flexible supercapacitors, and in radiotherapy applications [34,46,47,48]. Au@Mn_3_O_4_ nanoflower (30 nm)-shaped structures were prepared using a two-step method by growing Mn_3_O_4_ NPs onto Au seeds for T_1_-weighted MRI and PTT of breast cancer [49]. Previously, scarcely a report has been found on an electrochemical sensor to identify H_2_O_2_ produced from living cells using Au/MnO nanoparticles [50]. Therefore, it is essential to design biocompatible and functionalized Au and MnO hybrid composites using a simple synthesis method for cancer theranostic applications.

**Scheme 1:**
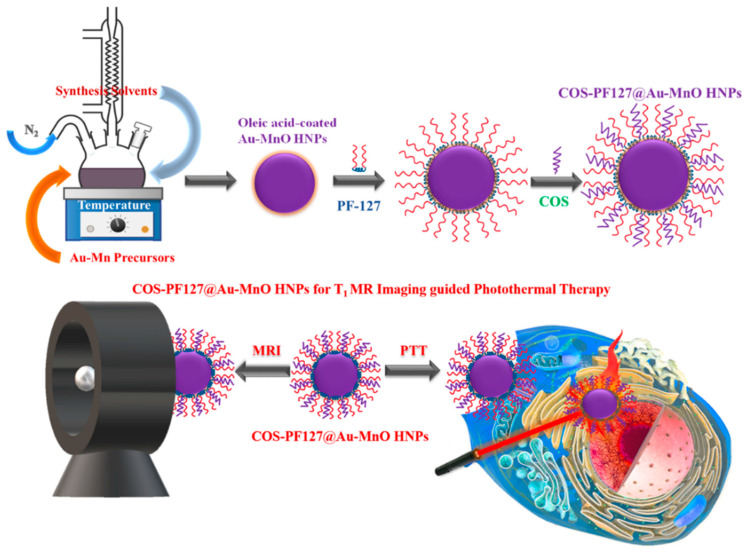
The graphical illustration of the synthesis process of COS-PF127@Au-MnO HNPs using the thermal decomposition method and their applications in T_1_ MR imaging-guided photothermal therapy.

In order to realize a promising theranostic agent and resolve the aforementioned serious toxicity issues of Gd- and Fe-based contrast agents, biocompatible plasmonic magnetic Au-MnO hybrid nanoparticles (HNPs) were prepared using a simple solution-based one-step synthesis method as shown in Scheme 1. Chitosan oligosaccharide (COS) and Pluronic^®^ F-127 polymers were used to improve the stability and solubility of the prepared oleic acid-coated Au-MnO HNPs in an aqueous medium owing to their numerous applications in biomedical and pharmaceutics [51,52]. The advanced characterization techniques confirmed the structural properties. In addition, the synthesized biocompatible multifunctional Au-MnO HNPs were effectively utilized in T_1_ MR imaging and photothermal therapy of breast cancer. 

## 2. Materials and Methods

### 2.1. Materials

Manganese (II) chloride tetrahydrate (MnCl_2_·4H_2_O, Adamas, Shanghai, China), sodium oleate (Adamas, Shanghai, China), hydrogen tetrachloroaurate (III) hydrate (HAuCl_4_·4H_2_O, Adamas, Shanghai, China), oleylamine (90%, Adamas, Shanghai, China), 1-octadecene (90%, General-reagent, Shanghai, China), Pluronic^®^ F-127 [poly(ethylene oxide)-poly(propylene oxide)-poly(ethylene oxide), PEO-PPO-PEO] (PF127, General-reagent, Shanghai, China), chitosan oligosaccharide (COS, Mw ≤ 2000, Macklin, China), oleic acid (Aladdin, Shanghai, China), fluorescein 5(6)-isothiocyanate (FITC, Aladdin, Shanghai, China), Calcein acetoxymethyl ester (Calcein AM, Beyotime, Shanghai, China), propidium iodide (PI, Beyotime, Shanghai, China), Dulbecco’s modified Eagle medium (DMEM, high glucose, Gibco, C11995), 3-(4,5-dimethylthiazol-2-yl)-2,5-diphenyl tetrazolium bromide (MTT, Beyotime, Shanghai, China), fetal bovine serum (FBS, Hyclone, Logan, UT, USA, SH30070.03), phosphate buffer saline (PBS, Hyclone, Logan, UT, USA, SH30256.FS). Trichloromethane (CHCl_3_), n-hexane, cyclohexane, ethanol, and acetone were purchased from Shuanglin chemicals, Hangzhou, China, and used as received.

### 2.2. Synthesis of Au-MnO HNPs

In order to prepare Au-MnO nanocomposites, manganese oleate was prepared by the previously reported method by replacing the precursor of iron with manganese and keeping the same conditions. Then, 30 mg of HAuCl_4_·4H_2_O was dispersed in 2 mL of oleylamine and sonicated for 10 min. In a three-necked flask, 0.4 mM of manganese oleate was introduced in a mixture of 1-octadecene (18 mL), oleic acid (1.9 mL), and oleylamine (1.9 mL) with continuous stirring under N_2_ flow at 120 °C for 20 min. The pretreated HAuCl_4_ solution was injected into the mixture then heated at 315 °C with a rate of 5 °C/min for 90 min. After that, the reaction solution was left to cool down at room temperature, and the product was precipitated using acetone. The solution was centrifuged (10,000 rpm, 10 min), and washed with acetone several times. Finally, the prepared oleic acid-coated Au-MnO HNPs were dispersed in cyclohexane (10 mL).

### 2.3. Preparation of COS-PF127-Au-MnO HNPs

The phase transfer of nanoparticles from organic to aqueous was achieved by the amphiphilic triblock copolymer Pluronic^®^ F-127 (PF127). Exactly 0.7 g of PF127 was dissolved in 70 mL of CHCl_3_ and magnetically stirred for 20 min to form a clear solution. Then, 1.5 mL of oleic acid-coated Au-MnO HNPs solution was injected gently into the above solution. After stirring for 4 h, 10 mL of Milli-Q water (18.25 MΩ·cm) was added into the solution and mixed via sonication. Afterward, CHCl_3_ was removed by rotary evaporation at 40 °C while the aqueous solution of PF127-coated Au-MnO HNPs accumulated. The 20 mg of chitosan oligosaccharide (COS) was added into the Au-MnO HNPs@PF127 solution and stirred overnight to accomplish COS coating. Furthermore, the final product of Au-MnO HNPs@PF127-COS was obtained by centrifugation (6000 rpm, 5 min) to remove surplus COS and then re-dispersed in Milli-Q water. The concentration of Au and Mn was determined by inductively coupled plasma mass spectrometry (ICP-MS), corresponding to the Mn: 610 μg/mL and Au: 287 μg/mL.

### 2.4. Characterizations

The transmission electron microscopy (TEM), high-resolution transmission electron microscopy (HRTEM), and energy dispersive spectroscopy (EDS) were carried out by JEM-2010 (JEOL, Tokyo, Japan). Scanning transmission electron microscopy (STEM) and EDS elemental mapping were conducted using Talos F200x (Thermo Fisher Scientific, Waltham, MA, USA). Inductive coupled plasma mass spectroscopy (ICP-MS) was accomplished by an Optima 2100 instrument (Perkin Elmer, Waltham, MA, USA) to measure the concentrations of Au and Mn in COS-PF127@Au-MnO. Dynamic light scattering (DLS) and Zeta potential of the samples were performed via the Zetasizer Nano series (Malvern, Malvern City, UK). The D8 Focus XRD diffractometer (Bruker, Karlsruhe, Germany) with Cu Kα radiation (λ = 0.154 nm) was employed to acquire information on the crystal structure of Au-MnO HNPs. Magnetic measurements were carried out using a vibrating sample magnetometer (VSM) option of a Model-9 PPMS (Quantum Design, San Diego, CA, USA). The UV–Vis absorption spectra (190–900 nm) of prepared HNPs were measured by U-3900 Spectrophotometer (Hitachi, Tokyo, Japan). The X-ray photoelectron spectroscopy (XPS) was performed using a K-ALPHA XPS (Thermo Fisher Scientific, Waltham, MA, USA). Instrument base pressure was 5 × 10^−9^ mbar using Al Kα X-ray source (hν = 100 eV–3000 eV), spot size ranged from 30–400 µm, and energy resolution was less than 0.5 eV corresponding to an Ag 3d5/2 peak of FWHM. 

### 2.5. Cell Culture and In Vitro Cytotoxicity Assay

The mouse breast cancer 4T1 cells (purchased from Shanghai Institutes for Biological Sciences, Shanghai, China) were maintained in DMEM medium supplemented with 10% FBS, 100 U/mL of penicillin, and 0.1 mg/mL streptomycin at 37 °C under 5% of carbon dioxide atmosphere. The cells were tested and authenticated negative for mycoplasma contamination. The cytotoxicity of Au-MnO@PF127-COS HNPs was investigated through a colorimetric methyl thiazolyl tetrazolium (MTT) assay with 4T1 cells. Briefly, the 4T1 cells were seeded into a 96-well plate at a density of 8 × 10^3^ cells per well and cultivated in 100 μL of DMEM medium for 24 h. The culture medium was replaced with a fresh culture medium for 24 h along with various concentrations of Au-MnO@PF127-COS HNPs such as 0, 40, 60, 80, 100, 120, 150, 200 μg/mL of Mn, including 0, 19, 29, 38, 48, 58, 71 and 96 μg/mL of Au. After washing with PBS, the medium was replaced with 15 μL of MTT solution (5 mg/mL) in a DMEM medium and incubated for another 4 h. After that, the supernatant was removed carefully, and MTT formazan crystals were dissolved by adding 150 μL of DMSO into each well. Later, the 96-well plate was placed on a shaker for 15 min. The absorbance value was measured at 570 nm by the SpectraMax Plus 384, Molecular Devices, San Jose, CA, USA. The cell viability of each well was evaluated through absorption measurement. 

### 2.6. MR Imaging and Relaxation Properties

The Au-MnO@PF127-COS HNPs dispersed in Milli-Q water with different concentrations Mn (0.34, 0.68, 1.36, 2.72, 5.44 mM) were used for MRI contrast performance and relaxation time testing. The 0.55 TMR imager (MeoMR60, Shanghai Niumag Corporation, Shanghai, China) was used to obtain the T_1_-weighted MR images (TR = 300 ms, TE = 18 ms, 256 × 256 matrices, 100 × 100 mm^2^ field of view, 2 mm slice thickness). The longitudinal (T_1_) relaxation time was measured via an inversion-recovery IR Sequence: P90(us) = 22, P180 (us) = 40.00, TD = 1024, SW (KHz) = 100, TR (ms) = 2000, RG1 = 20, RG2 = 3, NS = 2, DL1 (ms) = 1~2000, NTI = 30), TW = 3500 ms on the MesoMR23-060H-I imaging & analyzing system. The relaxivity value r_1_ was calculated through the linear fitting of 1/T_1_ (s^−1^) versus the Mn concentration (mM). The relaxation time measurement and MRI scan were performed at room temperature.

### 2.7. Cellular Uptake Studies

To carry out the cellular studies, fluorescein isothiocyanate (FITC) dye was used to label the Au-MnO@PF127-COS HNPs. FITC (50 μg/mL) was mixed with prepared HNPs and stirred overnight in the dark. Extra FITC was removed by centrifugation (×3) by washing with PBS, and the precipitate was re-dispersed in Milli-Q water for further use. The mouse mammary 4T1 cells were incubated with 1.5 mL of culture medium in a 35 mm glass-bottomed cell culture dish at a density of 1 × 10^5^ cells/mL overnight. Subsequently, the medium was replaced with a fresh DMEM and FITC-labeled Au-MnO@PF127-COS HNPs (Mn: 100 μg/mL, Au: 48 μg/mL) and incubated for a further 6 h. After 6 h, the cells were washed three times with PBS. Thereafter, 1 mL of paraformaldehyde was supplemented and placed in the dark for 10 min to fix cells. The paraformaldehyde was discarded, and the cells were washed with PBS three times. For staining cell plasma membrane DiI kit (red fluorescence) and for staining nucleus, DAPI (blue fluorescence) kits were used. Finally, the PBS-washed cells were captured by a confocal laser scanning microscope (CLSM, Nikon A1, Tokyo, Japan).

### 2.8. Photothermal Performance

The photothermal conversion was conducted by 200 μL of Au-MnO@PF127-COS HNPs with different concentrations of Au: 50, 100, 150, 200 mg/mL including concentrations of Mn: 106, 212, 318, 424 mg/mL. A continuous laser beam (Changchun Laser Optoelectronics Technology, Changchun, China) was applied with a wavelength of 808 nm to irradiate the sample vertically from top to bottom of the centrifuge tube for 10 min. In this experiment, 200 μL of Milli-Q water serves as a control. Further, the time-dependent temperature change was monitored every 30 s of an interval for 10 min using a thermal infrared camera (Fotric, Shanghai, China). Furthermore, the photothermal stability of Au-MnO@PF127-COS HNPs was measured by cycle irradiation. Firstly, 200 μL of Au-MnO@PF127-COS HNPs was exposed to a laser beam at 808 nm with 1.2 W/cm^2^ of intensity for 10 min. Later, the solution was cooled down to room temperature. For the photothermal conversion efficiency (η), 200 μL of Au-MnO@PF127-COS HNPs (Au: 150 μg/mL and Mn: 318 mg/mL) was exposed to the 1.5 W/cm^2^ intensity of a laser beam for 10 min. Thereafter, the irradiated solution was left at room temperature without a laser beam. The temperature change was monitored with a thermal infrared camera.

### 2.9. In Vitro Photothermal Therapy

The murine breast carcinoma 4T1 cells were seeded in a 96-well plate and cultivated for 24 h. Further after 12 h, the cells were treated with DMEM-diluted Au-MnO@PF127-COS HNPs at different concentrations of Au: 0, 25, 50, 100, 150, 200 μg/mL and Mn: 0, 53, 106, 212, 318, 424 mg/mL in the presence of 100 μL of the medium. After removing the culture medium, the cells were washed with PBS three times, then seeded with a fresh culture medium. Subsequently, the 4T1 cells were exposed to 808 nm laser irradiation for 10 min at an intensity of 1.5 W/cm^2^. After incubating for another 12 h, the cell viability was evaluated by MTT assay.

### 2.10. Live-Dead Cell Staining Experiments

The 4T1 cells were seeded in 35 mm glass-bottomed cell culture dishes overnight and then incubated with fresh DMEM containing Au-MnO@PF127-COS HNPs (Au: 150 μg/mL and Mn: 318 μg/mL), again overnight. After replacing the culture medium with fresh DMEM, the cells were treated with or without 808 nm laser irradiation (1.5 W/cm^2^) for 10 min and then incubated for 2 h. After that, the culture medium was removed carefully, and the cells were stained with both Calcein AM and propidium iodide (PI). The live and dead cells were observed through a confocal laser scanning microscope. 

## 3. Results and Discussions

### 3.1. Synthesis and Characterizations of Au-MnO HNPs

**Figure 1:**
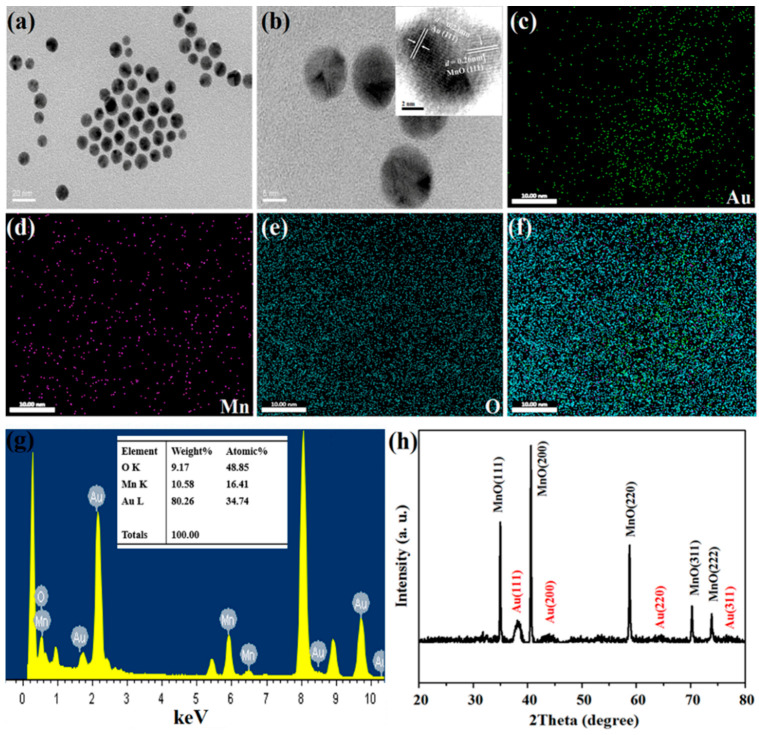
The structural characterization of Au-MnO HNPs. (**a**,**b**) low and high magnification TEM images (inset is a high resolution-TEM image of single hybrid nanoparticle), (**c**–**f**) STEM elemental color mapping images, (**g**) EDS spectra, and (**h**) the powder XRD pattern of prepared Au-MnO HNPs.

Small magnetoplasmonic Au-MnO hybrid nanoparticles were prepared by the facile one-step thermal decomposition method. Briefly, MnOl and HAuCl_4_ were selected as precursors because they have different reduction potentials. HAuCl_4_ has a fast reduction under mild conditions, resulting in the rapid formation of Au nanoparticles compared to MnOl. Therefore, seeds of Au NPs were firstly formed during synthesis, and then reduction of MnOl was carried out at high temperature to form oleic acid-coated Au-MnO hybrid nanocomposites with excellent dispersion in a nonpolar solvent. Initially, structural characterizations were investigated using transmission electron microscopy (TEM). Figure 1a,b shows the low and high magnification of Au-MnO HNPs with homogeneous size (8 nm), spherical shape, and dispersion. The indistinct lattice spacing and energy-dispersive X-ray spectroscopy (EDS) indicate the primary information of the nanocomposite construction. The lattice spacing of Au-MnO HNPs was obvious using a digital micrograph with an ABSF-filtered (average background subtraction filter) image, as revealed in the inset of Figure 1b. The lattice spacing values were 0.23 ± 0.002 and 0.26 ± 0.002 nm, consistent with the interplanar spacing of (111) in Au and (111) in MnO structures, respectively. Similar lattice spacing values were observed in four nanoparticles, as shown in Figure S1. In addition, scanning transmission electron microscopy (STEM) was performed to observe the in-depth information of the prepared hybrid structure. STEM mapping images show that the high density of Au elements (Figure 1c) is located closed packed, which is consistent with HRTEM. Moreover, Mn and oxygen elements are scattered over the Au, as shown in Figure 1d,e. The HRTEM and merged mapping images revealed the hybrid nature of synthesized Au-MnO HNPs. In addition, EDS spectra and atomic percentage (Figure 1g) further justify the successful formation of magnetoplasmonic Au-MnO HNPs without any impurity. X-ray diffraction (XRD) measurement was evaluated to understand the phase identification of the developed Au-MnO HNPs, as shown in Figure 1h. The major diffraction peaks (1 1 1), (2 0 0), (2 2 0), (3 1 1) and (2 2 2) correspond to face-centered cubic (fcc) MnO crystal structure and are well-matched with MnO (JCPDS#07-0230). The rest of the crystal planes are assigned to the face-centered cubic (fcc) structure of gold and are well-indexed with JCPDS#04-0784. The XRD, EDS, HRTEM, and STEM investigations exhibit good evidence for the formation of magnetoplasmonic Au-MnO HNPs with high purity. 

**Figure 2:**
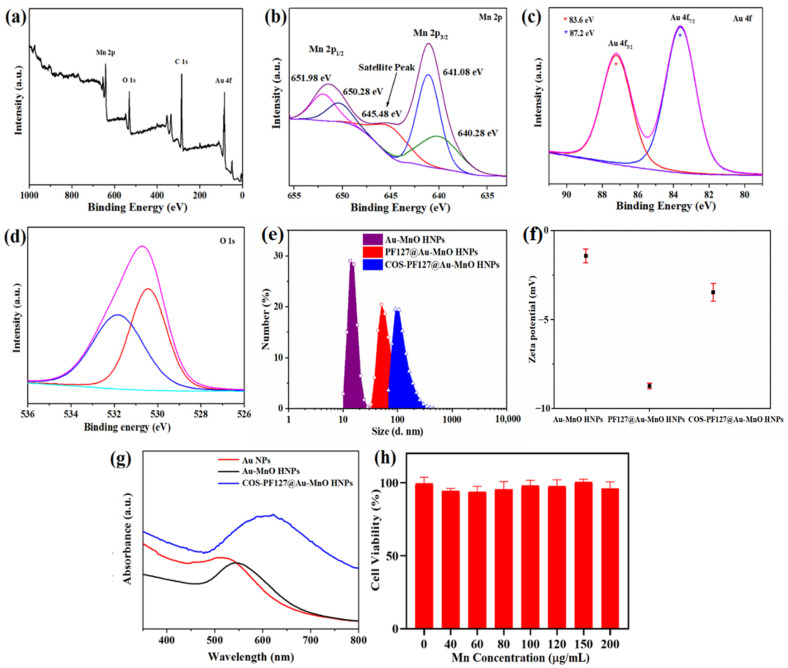
(**a**) XPS of a wide-scan (**b**) high-resolution Mn 2p, (**c**) O1s, and (**d**) Au 4f spectrum of synthesized Au-MnO HNPs. (**e**) Size distribution histogram, (**f**) zeta potential, and (**g**) UV–Vis spectra of fabricated Au-MnO nanocomposites with different coatings. (**h**) Cell cytotoxicity evolution was assessed by MTT assay using various concentrations of COS-PF127@Au-MnO HNPs with respect to Mn.

X-ray photoelectron spectroscopy (XPS) was used to understand the composition and chemical states of Mn and Au in Au-MnO hybrid nanocomposites. The wide-scan survey spectrum (Figure 2a) shows only signals from Mn, Au, C, and O elements, suggesting high purity consistent with STEM and EDS results. The higher resolution spectra of Mn 2p, Au 4f, and O 1s (Figure 2b–d) were fitted to multiple splitting peaks to identify the different oxidation states. The binding energies of 641.08 eV and 651.98 eV correspond to Mn 2p_3/2_ and Mn 2p_1/2_, respectively, which are consistent with the characteristics of MnO. Some mixed states (Mn^3+^ and Mn^4+^) are observed but were not as prominent as Mn^2+^, and a pronounced satellite peak was found at 645.48 eV, which does not exist for Mn^3+^ and Mn^4+^, indicating that the Mn element is in an oxidation state of +2 (Mn^2+^) [53]. The results are consistent with some previous reports. In addition, an energy separation between Mn 2p_3/2_ and Mn 2p_1/2_ levels is about 11 eV, further providing evidence of MnO presence in the prepared HNPs [54]. Figure 2c reveals the high-resolution XPS spectra of Au 4f, the binding energies located at 83.6 and 87.2 eV are assigned to Au 4f_7/2_ and Au 4f_5/2_, corresponding to elemental gold [55]. Moreover, the apparent peak of O 1s at about 530 eV (Figure 2d) exhibits the characteristic of MnO, which is associated with the Mn–O–Mn in Au-MnO HNPs. 

### 3.2. Surface Modification and Biocompatibility of Au-MnO HNPs

It is well known that surface modifications provide additional benefits such as enhanced biocompatibility, aqueous dispensability, and improved loading capacities for active targeting. The Food and Drug Administration (FDA)-approved triblock copolymer Pluronics F-127 and chitosan oligosaccharide (COS) polymers were grafted onto the surface of prepared Au-MnO HNPs. Both polymers have enriched utilization and a positive influence on biomedical applications. The average particle sizes and distributions, and zeta potentials of the prepared small nanoparticles were measured by dynamic light scattering. The hydrodynamic diameters of magnetoplasmonic Au-MnO, PF127@Au-MnO, and COS-coated PF127@Au-MnO HNPs are 14, 51, and 98 nm, respectively (Figure 2e). Their zeta potentials are −1.4, −8.5, and −3.5 mV, respectively, as shown in Figure 2f. The zeta potential values can be altered by increasing the concentration of the nanoparticles. A significant variation (positive to negative) is observed at the low concentration, and Au NPs showed concentration-dependent zeta potential [56,57]. However, the prepared COS-PF127@Au-MnO HNPs showed excellent colloidal stability as COS has a lower viscosity and higher water solubility than chitosan [52]. The particle size and negative potential of synthesized HNPs are promising for biomedical applications. The apparent variation in sizes and potentials indicates the successful coating of PF127 and COS. In addition, UV–Vis spectroscopy was used to investigate the influence of manganese and polymer coating on the absorption of Au. The characteristic peak at 520 nm was observed in the UV–Vis spectra, assigned to the plasmon for spherical Au NPs. However, the absorption intensity of Au is decreased, and a peak broadening with redshift was obtained after the addition of Mn. Interestingly, significant absorption was found after the COS-PF127 modification when compared with only Au-MnO nanoparticles (Figure 2g), indicating that the designed COS-PF127-coated Au-MnO HNPs are suitable for phototherapy. The biocompatibility of the prepared Au-MnO HNPs must be assessed before heading towards in vitro studies. COS-coated PF127@Au-MnO HNPs were selected to determine the cytotoxicity and for further studies because of their good water dispersion ability. The cell viability was measured using 3-(4,5-dimethyl-thiazol-2yl)-2,5-diphenyl tetrazolium bromide (MTT) assay. The MTT results (Figure 2f) demonstrate that the prepared polymer-coated HNPs do not show toxicity effects at different concentrations, showing good biocompatibility and appropriateness for their usage in biomedical applications. 

### 3.3. T_1_-MR Imaging and Relaxivity Properties of Au-MnO HNPs

**Figure 3:**
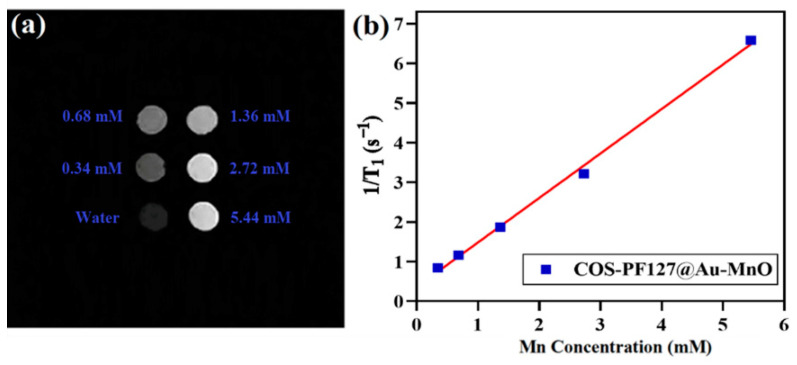
(**a**) T_1_-weighted MR images at room temperature and 0.55 T, and (**b**) longitudinal relaxation rate obtained using COS-PF127@Au-MnO HNPs aqueous suspensions as a function of Mn concentrations.

To assess the effectiveness of prepared HNPs as an MRI contrast agent, the MRI scan and relaxivity were carried out on a 0.55 T MRI scanner. Figure 3a shows the T_1_-weighted MR images of aqueous dispersed COS-PF127@Au-MnO HNPs at various concentrations of Mn obtained from the ICP test. The remarkable concentration-dependent brightening effect was observed, which was significant compared to water. The images indicate that the T_1_ signal intensity increases with the enhancement of Mn concentration. Moreover, the r_1_ relaxivity of prepared Au-MnO HNPs was calculated to be 1.2 mM^−1^ s^−1^ (Figure 3b) from the linear fitting of 1/T_1_(s^−1^) data against different concentrations of Mn. The calculated relaxivity value r_1_ is better than previously reported Mn-based nanocomposites depicted in the supplementary information (Table S1), demonstrating their potential for serving as T_1_ contrast agents. The high r_1_ relaxivity was attributed to the small size of prepared COS-PF127@Au-MnO HNPs [44]. 

### 3.4. Cellular Uptake of Au-MnO HNPs

**Figure 4:**
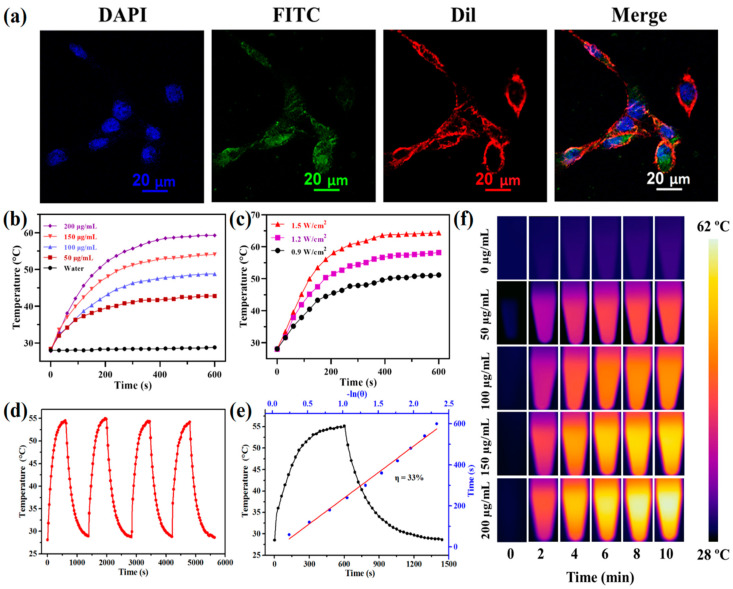
(**a**) Confocal microscopy images of intracellular distribution of COS modified PF127@Au-MnO HNPs for 6 h. Blue fluorescence shows 2-(4-Amidinophenyl)-6-indolecarbamidine dihydrochloride (DAPI) labeled nuclei, green fluorescence indicates FITC-labelled HNPs, red fluorescence represents cell membrane stained by 1,1’-dioctadecyl-3,3,3’,3’-tetramethylindocarbocyanine perchlorate (Dil), and merge is the combined effect to observed complete cell. (**b**) Photothermal conversion profile at various concentrations (50, 100, 150, and 200 µg mL^−1^) of prepared Au-MnO HNPs irradiated by 808 nm laser with 1.2 W/cm^2^ power intensity, (**c**) temperature rising graph of 200 µg mL^−1^ HNPs solution using variable laser power intensity (0.9, 1.2, and 1.5 W cm^−2^), (**d**) cyclic photothermal stability tests of 150 µg mL^−1^ HNPs solution under 1.2 W cm^−2^ laser irradiation, (**e**) heating and cooling curves of 150 µg mL^−1^ HNPs solution under 1.2 W cm^−2^ laser irradiation with linear fitting time data obtained from the cooling period and (**f**) photothermal images using an infrared camera. The scale bar is 20 μm.

The cell membrane is a complex system that consists of biomolecules and various proteins, possessing a generally negative charge of the plasma membrane. In vitro, cellular uptake is an interesting feature that must be analyzed to investigate the interaction of nanoparticles with biological medium, cellular entry, and therapeutic efficiency before in vivo experiments. Initially, fluorescein isothiocyanate (FITC) was adsorbed onto the surface of prepared COS-PF127@Au-MnO HNPs by stirring overnight. COS-modified PF127@Au-MnO HNPs exhibited a significant ability to encapsulate in the 4T1 tumor cells in 6 h (Figure 4a), as the biocompatible COS and PF127 possess potential anti-cancer targeting and drug delivery abilities [51,52], with the potential for admirable therapeutic outcomes. 

### 3.5. Photothermal Therapy of Au-MnO HNPs

Generally, Au nanoparticles show tunable localized surface plasmon resonance (LPSR) properties in the range of 450 nm–520 nm. However, introducing transition metals into Au NPs can produce a redshift in the LSPR bands towards the NIR region [58]. The photothermal properties and efficiency of synthesized Au-MnO HNPs were investigated using external stimuli from an 808 nm NIR laser. A thermal infrared camera was used to record the temperature variation profile and images. The concentration-dependent photothermal effect of aqueous dispersed COS-PF127@Au-MnO HNPs was observed under 808 nm laser with an intensity of 1.2 W cm^−2^ (Figure 4b). The temperature increased to 58 °C at 200 μg/mL after 6 min of irradiation, significantly higher than the control (27 °C). Interestingly, this temperature further increased to 65 °C (200 μg/mL) by increasing the laser power to 1.5 W cm^−2^ as shown in Figure 4c. Furthermore, promising photothermal stability of prepared HNPs was noticed by on/off NIR laser for four cycles, as the HNPs preserved the stable photothermal performance with their initial state (Figure 4d). In addition, the photothermal conversion efficiency (η) of Au-MnO HNPs was calculated to be 33% using a relevant formula from the cooling curve manifested in Figure 4e. The details of photothermal conversion efficiency calculations and efficiency compared with a similar system are mentioned in the supplementary information and Table S2, respectively. In order to understand the precise diagnosis and therapeutic effects, IR thermal images of NPs solutions with various concentrations irradiated under 1.2 W cm^−2^ were obtained. The thermal images (Figure 4f) suggest that COS-coated PF127@Au-MnO HNPs possessed exciting photothermal conversion capability and potential PTAs activated in the NIR region.

Encouraged by the remarkable temperature generated by prepared hybrid nanocomposites, an in vitro photothermal ablation experiment was conducted on 4T1 breast cancer cells using near-infrared light. A dramatically concentration-dependent decline in the cell viability was observed, as shown in the supplementary information (Figure S2). The cell viability decreased to less than 10% when designed HNPs (200 μg/mL) were incubated with tumorous 4T1 cells and irradiated with NIR laser, indicating the effectiveness of Au-MnO HNPs as a photothermal therapeutic agent.

**Figure 5:**
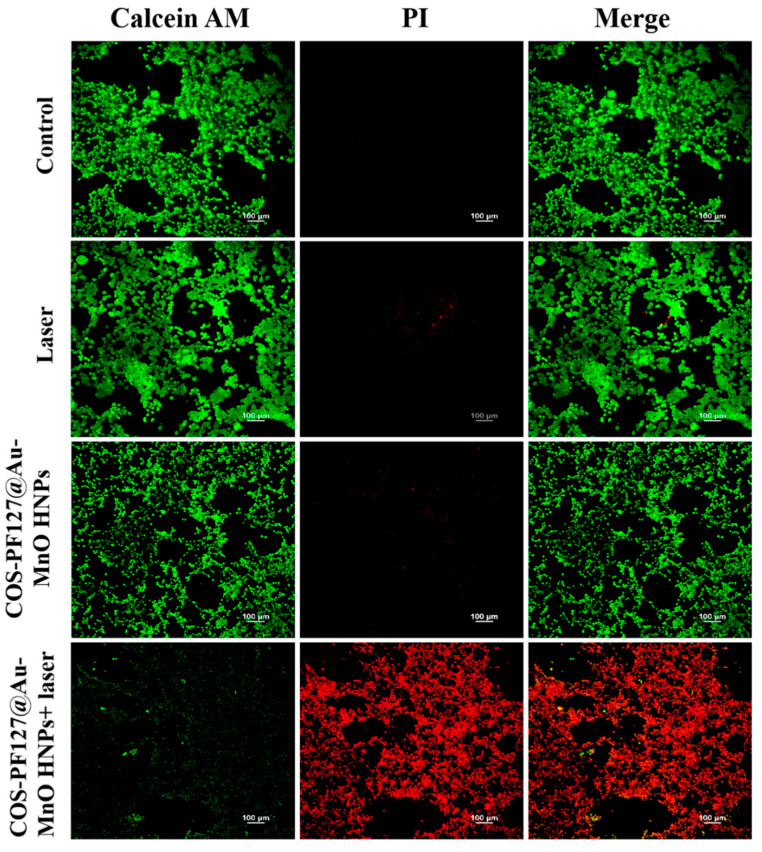
Fluorescent images of 4T1 cells stained with Live/Dead Double Stain Kit under different treatment groups such as PBS, laser, HNPs, and HNPs + laser. Green fluorescence indicates live cells which were stained by calcein-AM dye, red fluorescence indicates dead cells which were stained by propidium iodide (PI), and merge is the combined effect of live/dead cells. The scale bar is 100 μm.

The direct observation of the toxicity and the photothermal lethality effect of fabricated nanocomposites onto cancer cells, using live/dead double staining of 4T1 cells by calcein-AM/PI stain kit, was carried out. The significant viable cells (green fluorescence) were found in control and Au-MnO HNPs-treated groups, showing the negligible cytotoxicity of prepared hybrid HNPs, and these results are consistent with the MTT toxicity assay. Furthermore, a few cells died in the only laser-treated group. However, remarkable cell death (red fluorescence) was observed (Figure 5) when HNPs were irradiated under an 808 nm laser, indicating the excellent therapeutic performance of the designed Au-MnO HNPs.

## 4. Conclusions

In summary, a facile one-step thermal decomposition method was employed to prepare promising magnetoplasmonic Au-MnO hybrid nanocomposites for enhanced cancer theranostic applications. Structural and elemental investigation showed the controlled synthesis, homogeneous size, and morphology. Zeta potential and UV–Vis results further indicated the successful surface modification of Au-MnO by PF127 and COS polymers. The polymer coating improved the biocompatibility and aqueous dispersion of designed Au-MnO HNPs. A significant enhancement was observed in T_1_ MR imaging due to paramagnetic Mn ions, which are a good alternative to Gd-based metal chelates. Meanwhile, NIR-I emission of the nano-platform stimulated by 808 nm light provided effective photothermal killing efficacy against 4T1 breast cancer cells. As a result, the designed biocompatible COS-PF127@ Au-MnO HNPs demonstrated a potential cancer theranostic system.

## Data Availability

The data are available upon reasonable request from the corresponding author.

## Supplementary Materials

The following supporting information can be downloaded at: https://www.mdpi.com/article/10.3390/nano12081370/s1, Figure S1: High resolution-TEM images of multiple Au-MnO hybrid nanoparticles; Figure S2: Viability of 4T1 cells treated with varying concentrations of COS-PF127-Au-MnO QDs with 808 nm laser irradiation (10 min); Table S1: Relaxivity comparison data of manganese oxide-based nanoparticles; Table S2: Comparison of photothermal conversion efficiency of various nanocomposites. References [59,60,61,62,63,64,65,66,67,68,69,70,71,72] are cited in the supplementary materials.
