# Supplementary material for: Facile Synthesis of Multifunctional Magnetoplasmonic Au-MnO Hybrid Nanocomposites for Cancer Theranostics
Source: Nanomaterials (Basel). 2022 Apr 16;12(8):1370. doi: 10.3390/nano12081370 (PMC9027802; doi:10.3390/nano12081370)
Supplement: Supplementary file 1 [file nanomaterials-12-01370-s001.zip › nanomaterials-1630273-supplementary.pdf]

Supplementary Materials

Facile Synthesis of Multifunctional Magnetoplasmonic Au-MnO Hybrid Nanocomposites for Cancer Theranostics

Cong Tian [1,2], Zhe Tang [1,2], Yike Hou [1,2], Asim Mushtaq [1,2], Shafaq Naz [3], Zhangsen Yu [4], Jabeen Farheen [1,2], Muhammad Zubair Iqbal [1,2,*] and Xiangdong Kong [1,2,*]

1 Institute of Smart Biomedical Materials, School of Materials Science and Engineering, Zhejiang Sci-Tech University, Hangzhou 310018, China; 201920301046@mails.zstu.edu.cn (C.T.); 202030302183@mails.zstu.edu.cn (Z.T.); 202110301007@mails.zstu.edu.cn (Y.H.); asim_college@yahoo.com (A.M.); jabeenfarheen87@zstu.edu.cn (J.F.)

2 Zhejiang-Mauritius Joint Research Center for Biomaterials and Tissue Engineering, Hangzhou 310018, China

3 Department of Mathematics, University of Gujrat, Hafiz Hayat Campus, Gujrat 50700, Pakistan; drshafaq.naz@uog.edu.pk

4 Laboratory of Nanomedicine, Medical Science Research Center, School of Medicine, Shaoxing University, Shaoxing 312000, China; yzs@usx.edu.cn

* Correspondence: zubair@zstu.edu.cn (M.Z.I.); kongxd@zstu.edu.cn (X.K.)

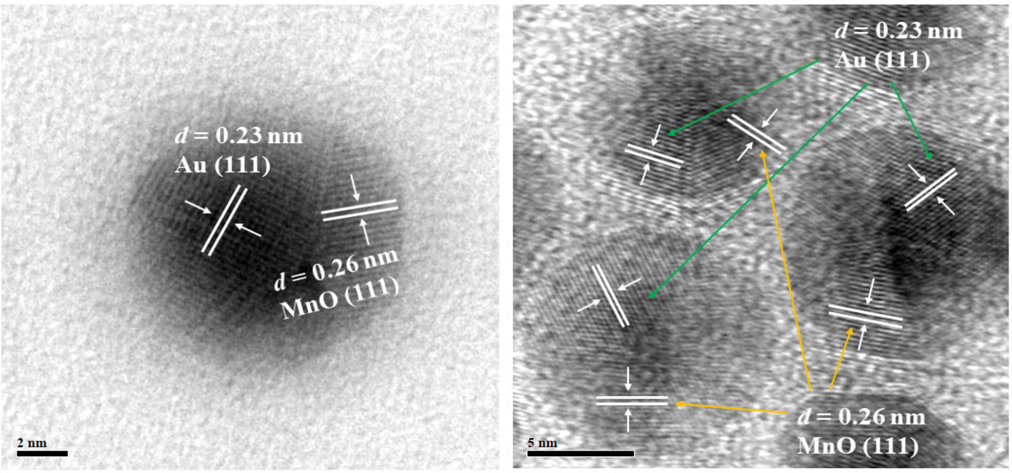

Figure S1. High resolution-TEM images of multiple Au-MnO hybrid nanoparticles.

Table S1: Relaxivity comparison data of manganese oxide-based nanoparticles.

Name	Core material	B_0 (T)	r_1 ($mM^{-1}S^{-1}$)	Ref.
COS-PF127@Au-MnO HNPs	Au	0.55	1.2	Current data
MnO@PEG-phospholipids	MnO	3	0.37	[1]
MnO@PEG-phospholipids	MnO	3	0.18	[1]
MnO@PEG-phospholipids	MnO	3	0.13	[1]
MnO@PEG-phospholipids	MnO	3	0.12	[1]
Au@MnO	Au		0.224	[2]
MnO@trioctylphosphine oxide - silica (poly(ethylene oxide F127 micelles))	MnO	1.5	1.17	[3]
MnO@trioctylphosphine oxide - silica (poly(ethylene oxide F127 micelles))	MnO	1.5	0.976	[3]
MnO@PLGA	MnO	4	0.21	[4]
MnO@mSiO$_2$-Ir-PEG	MnO	3	0.17	[5]

MnO@PEG2000-PE-DC cholesterol-DOPE	MnO	7	1.17	[6]
Mn_3O_4@SiO_2-PEG-aptamerAS411	Mn_3O_4	0.5	0.53	[7]
Mn_3O_4@SiO_2(PEG)FA	Mn_3O_4	0.5	0.49	[8]
MnO@SiO_2-PEG/NH_2	MnO	3	0.47	[9]

The photothermal conversion efficiency

The photothermal conversion efficiency of Au-MnO HNPs was calculated according to previous reports.

The total energy balance of this system as following equation:

$$\sum_i m_i C_{p,i} \frac{dT}{dt} = Q_{QDs} + Q_s - Q_{loss} \tag{S1}$$

where m and C_p are the mass and heat capacity, respectively. The suffix "i" of m and C_p refers to solvent (water) or dispersed matter (HNPs). T is the solution temperature. Q_{QDs} is the photothermal energy generated by Au-MnO HNPs. Qs is the heat associated with the light absorbed by solvent. Q_{loss} is thermal energy lost to the surroundings. Q_{QDs} can be obtained from the following equation:

$$Q_{QDs} = \mathrm{I}(1 - 10^{-A_\lambda})\eta \tag{S2}$$

where I is the laser power intensity, A_λ is the absorbance of Au-MnO HNPs at the wavelength of 808 nm in aqueous solution, and η is the photothermal conversion efficiency of Au-MnO HNPs which means the ratio of absorbed light energy converting to thermal energy.

In the situation of heating pure water, the heat input is equal to the heat output at the maximum steady-statue temperature, so the equation can be:

$$Q_{loss} = hA\Delta T = hA(T - T_{surr}) \tag{S3}$$

Where h is the heat transfer coefficient, A is the surface area of the container, T is the temperature of solution. T_{surr} is the ambient surrounding temperature.

As it to the experiment of Au-MnO HNPs dispersion, the heat inputs are the heat generated by nanoparticles (Q_{QDs}) and the heat generated by water (Q_s), which is equal to the heat output at the maximum steady-statue temperature. As $\Delta T_{max,\ QDs} = T_{max,\ QDs} - T_{surr}$, so the equation can be written as:

$$Q_{QDs} + Q_s = Q_{loss} = hA\Delta T_{max,\ QDs} \tag{S4}$$

Where $T_{max,\ QDs}$ is the temperature of the Au-MnO HNPs dispersion at the maximum temperature steady-state.

The photothermal conversion efficiency (η) can be expressed as following equation:

$$\eta = \frac{hA(\Delta T_{max,\ QDs} - \Delta T_{max,\ water})}{I(1 - 10^{-A_\lambda})} \tag{S5}$$

In this equation, only hA is unknown and the θ will introduce to calculate the value of hA. As θ is equal to ΔT / $\Delta T_{max,\ QDs}$. Here, hA can be obtained by equation:

$$hA = \frac{m\ C_P}{\tau_S} \tag{S6}$$

In which m and C_P are the mass and thermal capacity of the sample, respectively, and τ_s is the system time constant.

$$t = -\tau_s \ln(\theta) = -\tau_s \ln\left(\frac{T - T_{surr}}{T_{max,\ QDs} - T_{surr}}\right) \quad (S7)$$

t is the time of the cooling process after irradiation. τ_s is calculated by the slope of fitted line of time data against $-ln(\theta)$.

In this work, τ_s was calculated as 256.4 s. The values of m and C_P are 0.2 g and 4.2 J/(g·°C), respectively. Subsequently, 'hA' is calculated as 0.0033 mW/°C, and $A_{808\ nm}$, *I*, and $T_{max,QDs} - T_{surr}$ are measured as 0.10, 1.2 W/cm^2, and 27.7 °C, respectively. Above all, the photothermal conversion efficiency was calculated as 33 %.

Table S2. Comparison of photothermal conversion efficiency of various nanocomposites.

Materials	Laser Wavelength (nm)	Laser Power Intensity (W/cm^2)	Efficiency (%)	Reference
Au-MnO QDs	808	1.2	33	This work
Gold Nanoshell@ Mesoporous Silica Nanorod	808	8	17	[10]
Au-Fe_2C JNPs	808	1	30.2	[11]
MnO_2-mSiO_2@Au NPs	808	1.5	25.38	[12]
Cu_9S_5 NPs	980	0.51	25.7	[13]
$Cu_{2-x}Se$ nanocrystals	800	2	22	[14]
GNR@SiO_2@MnO_2	1064	0.4	27.47	[15]
$Cu_{2-x}Te$ nanocubes	808	2.02	25.68	[16]

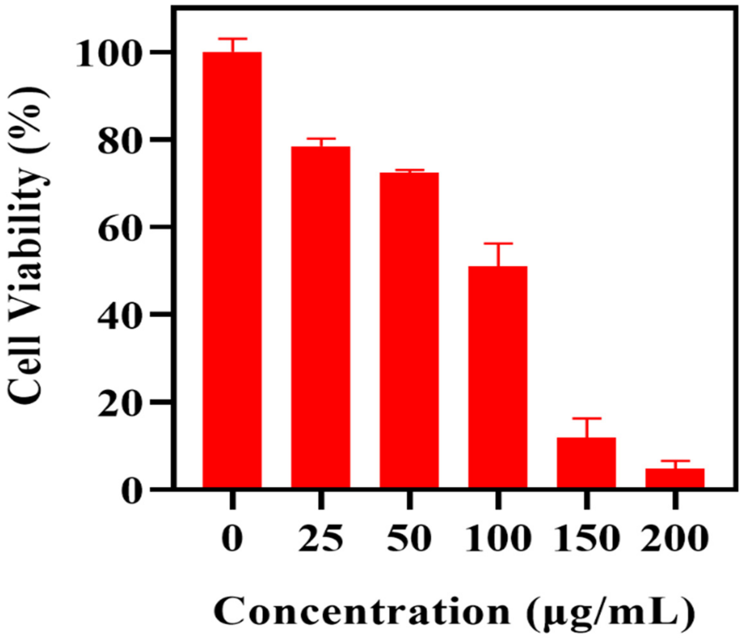

Figure S2. Viability of 4T1 cells treated with varying concentrations of COS-PF127-Au-MnO QDs with 808 nm laser irradiation (10 min).

References

1. Na, H.B.; Lee, J.H.; An, K.; Park, Y.I.; Park, M.; Lee, I.S.; Nam, D.H.; Kim, S.T.; Kim, S.H.; Kim, S.W.; et al. Development of a T1 contrast agent for magnetic resonance imaging using MnO nanoparticles. *Angewandte Chemie (International ed. in English)* **2007**, *46*, 5397-5401, doi:10.1002/anie.200604775.
2. Schladt, T.D.; Shukoor, M.I.; Schneider, K.; Tahir, M.N.; Natalio, F.; Ament, I.; Becker, J.; Jochum, F.D.; Weber, S.; Köhler, O.; et al. Au@MnO Nanoflowers: Hybrid Nanocomposites for Selective Dual Functionalization and Imaging. *Angewandte Chemie International Edition* **2010**, *49*, 3976-3980, doi:https://doi.org/10.1002/anie.200906689.
3. Hsu, B.Y.W.; Ng, M.; Zhang, Y.; Wong, S.Y.; Bhakoo, K.; Li, X.; Wang, J. A Hybrid Silica Nanoreactor Framework for Encapsulation of Hollow Manganese Oxide Nanoparticles of Superior T1 Magnetic Resonance Relaxivity. *Advanced Functional Materials* **2015**, *25*, 5269-5276, doi:https://doi.org/10.1002/adfm.201501269.
4. Bennewitz, M.F.; Lobo, T.L.; Nkansah, M.K.; Ulas, G.; Brudvig, G.W.; Shapiro, E.M. Biocompatible and pH-sensitive PLGA encapsulated MnO nanocrystals for molecular and cellular MRI. *ACS Nano* **2011**, *5*, 3438-3446, doi:10.1021/nn1019779.
5. Peng, Y.K.; Lai, C.W.; Liu, C.L.; Chen, H.C.; Hsiao, Y.H.; Liu, W.L.; Tang, K.C.; Chi, Y.; Hsiao, J.K.; Lim, K.E.; et al. A new and facile method to prepare uniform hollow MnO/functionalized $mSiO_2$ core/shell nanocomposites. *ACS Nano* **2011**, *5*, 4177-4187, doi:10.1021/nn200928r.
6. Howell, M.; Mallela, J.; Wang, C.; Ravi, S.; Dixit, S.; Garapati, U.; Mohapatra, S. Manganese-loaded lipid-micellar theranostics for simultaneous drug and gene delivery to lungs. *J Control Release* **2013**, *167*, 210-218, doi:10.1016/j.jconrel.2013.01.029.
7. Hu, H.; Dai, A.; Sun, J.; Li, X.; Gao, F.; Wu, L.; Fang, Y.; Yang, H.; An, L.; Wu, H.; et al. Aptamer-conjugated Mn_3O_4@SiO_2 core–shell nanoprobes for targeted magnetic resonance imaging. *Nanoscale* **2013**, *5*, 10447-10454, doi:10.1039/C3NR03490A.
8. Yang, X.; Zhou, Z.; Wang, L.; Tang, C.; Yang, H.; Yang, S. Folate conjugated Mn_3O_4@SiO_2 nanoparticles for targeted magnetic resonance imaging in vivo. *Materials Research Bulletin* **2014**, *57*, 97-102, doi:https://doi.org/10.1016/j.materresbull.2014.05.023.
9. Schladt, T.D.; Koll, K.; Prüfer, S.; Bauer, H.; Natalio, F.; Dumele, O.; Raidoo, R.; Weber, S.; Wolfrum, U.; Schreiber, L.M.; et al. Multifunctional superparamagnetic MnO@SiO_2 core/shell nanoparticles and their application for optical and magnetic resonance imaging. *Journal of Materials Chemistry* **2012**, *22*, 9253-9262, doi:10.1039/C2JM15320C.
10. Zhao, R.; Han, X.; Li, Y.; Wang, H.; Ji, T.; Zhao, Y.; Nie, G. Photothermal Effect Enhanced Cascade-Targeting Strategy for Improved Pancreatic Cancer Therapy by Gold Nanoshell@Mesoporous Silica Nanorod. *ACS Nano* **2017**, *11*, 8103-8113, doi:10.1021/acsnano.7b02918.
11. Ju, Y.; Zhang, H.; Yu, J.; Tong, S.; Tian, N.; Wang, Z.; Wang, X.; Su, X.; Chu, X.; Lin, J.; et al. Monodisperse Au-Fe_2C Janus Nanoparticles: An Attractive Multifunctional Material for Triple-Modal Imaging-Guided Tumor Photothermal Therapy. *ACS Nano* **2017**, *11*, 9239-9248, doi:10.1021/acsnano.7b04461.
12. Wang, S.; You, Q.; Wang, J.; Song, Y.; Cheng, Y.; Wang, Y.; Yang, S.; Yang, L.; Li, P.; Lu, Q.; et al. MSOT/CT/MR imaging-guided and hypoxia-maneuvered oxygen self-supply radiotherapy based on one-pot MnO_2-$mSiO_2$@Au nanoparticles. *Nanoscale* **2019**, *11*, 6270-6284, doi:10.1039/c9nr00918c.
13. Tian, Q.; Jiang, F.; Zou, R.; Liu, Q.; Chen, Z.; Zhu, M.; Yang, S.; Wang, J.; Wang, J.; Hu, J. Hydrophilic Cu_9S_5 nanocrystals: a photothermal agent with a 25.7% heat conversion efficiency for photothermal ablation of cancer cells in vivo. *ACS Nano* **2011**, *5*, 9761-9771, doi:doi: 10.1021/nn203293t.
14. Hessel, C.M.; Pattani, V.P.; Rasch, M.; Panthani, M.G.; Koo, B.; Tunnell, J.W.; Korgel, B.A. Copper selenide nanocrystals for photothermal therapy. *Nano Lett* **2011**, *11*, 2560-2566, doi:10.1021/nl201400z.
15. He, T.; Jiang, C.; He, J.; Zhang, Y.; He, G.; Wu, J.; Lin, J.; Zhou, X.; Huang, P. Manganese-Dioxide-Coating-Instructed Plasmonic Modulation of Gold Nanorods for Activatable Duplex-Imaging-Guided NIR-II Photothermal-Chemodynamic Therapy. *Adv Mater* **2021**, *33*, e2008540, doi:10.1002/adma.202008540.
16. Poulose, A.C.; Veeranarayanan, S.; Mohamed, M.S.; Aburto, R.R.; Mitcham, T.; Bouchard, R.R.; Ajayan, P.M.; Sakamoto, Y.; Maekawa, T.; Kumar, D.S. Multifunctional $Cu_{2-x}Te$ Nanocubes Mediated Combination Therapy for Multi-Drug Resistant MDA MB 453. *Sci Rep* **2016**, *6*, 3591, doi:10.1038/srep35961.
